# Follicular stage-dependent regulation of apoptosis and steroidogenesis by prohibitin in rat granulosa cells

**DOI:** 10.1186/1757-2215-6-23

**Published:** 2013-04-08

**Authors:** Qi Wang, Arthur Leader, Benjamin K Tsang

**Affiliations:** 1 Department of Cellular & Molecular Medicine, University of Ottawa, Ottawa, Ontario, K1H 8L6Canada; 2 Chronic Disease Program, Ottawa Hospital Research Institute, The Ottawa Hospital–General Campus, 501 Smyth Road, Ottawa, Ontario, Mail Box #511, K1H 8L6, Canada; 3 Reproductive Biology Unit, Department of Obstetrics and Gynaecology, University of Ottawa, Ottawa, Ontario, K1H 8L6, Canada; 4 Ottawa Fertility Centre, Ottawa, Ontario, K2C 3V4, Canada; 5 WCU Biomodulation Major, Department of Agricultural Biotechnology, College of Agriculture and Life Sciences, Seoul National University, Seoul, Republic of Korea

**Keywords:** Prohibitin, Apoptosis, Steroidogenesis

## Abstract

**Background:**

Follicular growth and atresia are tightly regulated processes, which involve the participation of endocrine, autocrine and paracrine factors at the cellular level. Prohibitin (PHB) is a multifunctional intracellular protein playing an important role in the regulation of proliferation, apoptosis and differentiation. Here we examined the expression of PHB and its regulation by FSH *in vitro* and studied the role of PHB in the regulation of apoptosis and steroidogenesis in response to the apoptosis inducer staurosporine (STS) and to FSH, respectively.

**Methods:**

Undifferentiated and differentiated granulosa cells were collected from diethylstilbestrol (DES)- and equine chronic gonadotropin (eCG)-primed immature rats, respectively and then cultured with various treatments (FSH, adenovirus infection, STS) according to experimental design. The apoptosis rate, the production of estradiol and progesterone, and the expression of distinct proteins (PHB, caspase-3, phospho- and total Akt) were assessed.

**Results:**

PHB is anti-apoptotic and its action is dependent on the differentiated state of the granulosa cells. Data from gain- and loss-of-function experiments demonstrate that PHB inhibited STS-induced caspase-3 cleavage and apoptosis in undifferentiated granulosa cells, but was ineffective in differentiated cells. In contrast, PHB suppresses FSH-induced steroidogenesis and this response is evident irrespective of the differentiated state of granulosa cells.

**Conclusion:**

These findings suggest that PHB regulates granulosa cell apoptosis and steroidogenesis in a follicular stage-dependent manner and that the dysregulation of PHB expression and action may be relevant to ovarian dysfunction.

## Background

The destiny of the growing follicles (ovulation or atresia) is dependent on the fate of the cells within them (proliferation, differentiation or apoptosis) and is tightly regulated by endocrine, autocrine and paracrine factors [1]. During follicular development, a large number of follicles undergo atresia, a process tightly controlled by the cross-talk of cell death and survival signals [1,2]. The dominant follicles continue to develop to preovulatory stages, producing appreciable amounts of steroid hormones which are critical for the reproductive cycle and successful ovulation [3,4].

Prohibitin (PHB) is a multifunctional protein highly conserved in various species, with identical amino acid sequences in mouse and rat and only one residue differing from that in human [5]. It is present in multiple cellular compartments, including nucleus, mitochondria, plasma membrane and lipid droplets, as well as in the circulation [6-10]. The subcellular localization of PHB may contribute to its diverse functions in the regulation of proliferation, apoptosis, senescence and differentiation [11-14]. For example, mitochondrial PHB facilitates the maintenance of mitochondrial morphology and stabilizes newly synthesized mitochondrial enzymes [15,16]. Nuclear PHB has been implicated in the regulation of gene expression by interacting with various transcriptional factors, such as E2F, p53 and estrogen receptor α (ERα) [7,17,18].

PHB is widely expressed in the ovary and is anti-apoptotic during staurosporine (STS)- and ceramide-induced apoptosis in undifferentiated granulosa cells [19-21]. However, it is unknown whether PHB performs similar roles during follicular development. Although recent data indicated that silencing of PHB induced granulosa cell shape changes [22] and PHB suppressed steroidogenesis in undifferentiated granulosa cells [23], our knowledge on the role and contribution of PHB to granulosa cell differentiation is incomplete.

In this study, we first examined the expression of PHB and their regulation by FSH *in vitro*. Using differentiated and undifferentiated granulosa cells from distinct stages of follicular development, we compared its responsiveness to the apoptosis inducer STS and to FSH, a differentiation inducer, and also examined the role of PHB by gain- and loss-of function experiments. We also studied if the roles of PHB in the regulation of apoptosis and steroidogenesis are follicular stage-dependent.

## Materials and methods

### Antibodies and reagents

Cell culture media (M199), fetal bovine serum (FBS), penicillin and streptomycin, L-glutamine, sodium pyruvate and trypsin were purchased from Invitrogen (Burlington, Canada). HEPES, Hoechst 33258, equine chronic gonadotropin (eCG), and diethylstilbestrol (DES) were purchased from Sigma (St. Louis, MO). Recombinant human FSH was purchased from National Hormone and Peptide Program (Harbor-UCLA Medical Center, Torrance, CA). Anti-caspase-3 antibody (recognizing both intact and active caspase-3), anti-phospho-Akt (S473) and anti-Akt antibodies were purchased from Cell signaling (Danvers, MA), anti-PHB and anti-β-Actin antibodies were from Abcam (Cambridge, MA). Horseradish peroxidase-conjugated secondary antibodies and reagents for SDS-PAGE were supplied by Bio-Rad (Mississauga, Canada). Enhanced chemiluminescent (ECL) reagent was from Thermo Fisher Scientific (Rockford, IL). Adenoviral-PHB, shPHB and their control particles were obtained from Dr. Winston Thompson (Morehouse School of Medicine, Atlanta). QIAShredder and RNeasy mini kit were purchased from QIAGEN (Mississauga, Canada). Random decamer primers were from Ambion (Austin, TX). Ribonuclease inhibitor and dNTP were from Fermentas (Burlington, Canada). Moloney murine leukemia virus reverse transcriptase was from Promega (Madison, WI). PCR primers were from Invitrogen. All chemical inhibitors were purchased from Calbiochem (Gibbstown, NJ). All other chemicals were of the highest analytical grade available from Sigma.

### Animal preparation

Twenty one days old Sprague–Dawley rats (Charles River, Montreal, Canada) were maintained on 12-h light, 12-h dark cycles and given food and water *ad libitum*. All procedures were carried out in accordance with the Guidelines for the Care and Use of Laboratory Animals, Canadian Council on Animal Care, and were approved by the University of Ottawa and the Ottawa Hospital Research Institute Animal Care Committee.

### Primary culture of rat granulosa cells and adenoviral infection

Granulosa cells from eCG-primed immature rats (10 IU, 48h, s.c.; considered as differentiated granulosa cells) and DES-injected control rats (1 mg/day for 3 consecutive days, s.c.; considered as undifferentiated granulosa cells) were pre-incubated with 6 mM EGTA and 0.5 M sucrose [24] and were released by follicular puncture with a 26.5-gauge needle, washed and centrifuged (900 × g, 5 min). Cell clumps and oocytes were removed by filtering the cell suspensions through a 40-μm nylon cell strainer (BD Biosciences). The viability of granulosa cells was determined by trypan blue exclusion. Granulosa cells (0.9 × 10^6^ per well in 6-well plate) were plated overnight in M199 with 10% FBS under a humidified atmosphere of 95% air and 5% CO_2_. After culture overnight in serum-free medium, granulosa cells were treated with FSH (0–200 ng/ml) or STS (1 μM) for a designated duration.

For adenoviral infection, granulosa cells were cultured in serum-free M199 medium containing adenoviral particles for 24 h followed by medium change. Multiplicity of infection (MOI) and duration of infection are detailed in the figures. Equal amounts of adenovirus in each experimental group were achieved by adjusting with an appropriate amount of adenoviral-LacZ (negative control for adenoviral-PHB) or adenoviral-shNeg (negative control for shPHB).

### RT-PCR

Total RNA of granulosa cells was extracted according to the manufacturer’s instruction, using the QIAGEN RNeasy Mini kit. Two hundred ng total RNA were used to reverse transcribe cDNAs and the mRNA abundance of target genes was analyzed by PCR. The PHB primers used for amplification were a 5^′^ forward primer (5^′^-TGGCAGCCTGAGTAGACCTT-3^′^) and a 3^′^ reverse primer (5^′^-TCACGGTTAAGAGGGAATGG-3^′^). The p450scc primers were a 5^′^ forward primer (5^′^-ACCCTGAGTCCCAGCGGTTC-3^′^) and a 3^′^ reverse primer (5^′^-CACCCC-TCCTGCCAGCATCT-3^′^). The aromatase primers were a 5^′^ forward primer (5^′^-TGGTCCCG-GAAACTGTGCCT-3^′^) and a 3^′^ reverse primer (5^′^-CCACGCTTGCTGCCGAATCT-3^′^). The actin primer were (5^′^-CGTCCACCCGCGAGTACAAC-3^′^) and a 3^′^ reverse primer (5^′^- GCCT-CTCTTGCTCTGGGCCT-3^′^). The thermal cycling conditions were comprised of an initial denaturation step at 95 C for 10 min followed by 30 cycles amplification for PHB, p450scc and aromatase (20 cycles for actin) at 95 C for 30 sec, 55 C for 30 sec, and 72 C for 30 sec. The PCR products were subjected to 2% ethidium bromide-containing agarose gel and visualized under UV light.

### Protein extraction and western blot

At the end of the culture period, floating cells and attached cells (recovered by 0.05% trypsin treatment) were pooled and centrifuged (1000 × g, 10 min). For protein extraction, cell pellets were resuspended in a lysis buffer (PBS, pH 7.4) containing NaCl (150 mM), SDS (0.1%), sodium deoxycholate (0.5%), Nonidet P-40 (1%), and the protease inhibitor cocktail (Sigma) and kept on ice for 30 min. Cell lysates were sonicated and centrifuged (12,000 × g, 5 min, 4°C) to remove insoluble material. Supernatant was recovered and stored at −20°C until further processing. Protein concentrations in each sample were determined by the Bradford assay (Bio-Rad Laboratories). Twenty μg of protein of cell lysates were subjected to SDS-PAGE with 4.5% stacking and 15% separating gels. Proteins were electrophoretically transferred onto nitrocellular membrane (NC, Bio-Rad), blocked at room temperature with 5% skim milk in TBST [0.05% Tween-20 in Tris (10 mM) and NaCl (0.15 M), pH7.4 (TBS)] for 1 h and then incubated overnight at 4°C with diluted primary antibodies (1:1000) in TBST with constant agitation. The membranes were then treated with a secondary antibody (1:2000 to 1:10,000 based on different primary antibody). After washing three times with TBST, immunoreactive bands were visualized with ECL according to the manufacturer’s instruction. Intensity of bands of the exposed X-ray film was determined by densitometrically scanned, quantified, using AlphaEaseFC (Alpha Innotech, CA) and normalized with β-Actin.

### Assessment of apoptosis

Apoptotic cells were identified morphologically by Hoechst-33258 (bisBenzimide, Sigma) staining as previously reported [25]. At the end of the culture period, suspended cells were collected by centrifugation and attached cells were trypsinized. The two cell fractions were then pooled, pelleted, and suspended in 10% phosphate-buffered formalin containing Hoechst 33258 (6.25 μg/ml; room temperature, 2 h), Cells were then spotted on slides and assessed for typical apoptotic nuclear morphology. To quantify the number of apoptotic cells, healthy and apoptotic cells were counted (counter was “blinded to sample identity”) and the apoptotic cells were expressed as a percentage of total cells. A minimum of 400 cells were counted in each treatment group.

### Steroids secretion analysis

Spent medium from granulosa cell cultures were collected, centrifuged (900 × g, 5 min) and kept in −80°C for hormone analysis. 17β-estradiol and progesterone concentrations in spent medium were measured using enzyme immunoassay kit (EIA; Enzo Life Sciences, Farmingdale, NY) according to the manufacturer’s instruction. The detection limitation of estradiol was 28 pg/ml, and the intra- and inter-assay coefficients of variation were 8 and 6%, respectively. The detection limitation of progesterone was 8.5 pg/ml, and the intra- and inter-assay coefficients of variation were 7 and 6%, respectively.

### Statistical analysis

All data were analyzed using GraphPad Prism 5.0 statistical software (San Diego, CA). Results are expressed as mean ± SEM of at least three independent experiments as detailed in the figures. One-way and two-way ANOVA were used to assess the effects and interactions of one or two variables and multiple comparisons were achieved by a Bonferroni *post hoc* test. Significant difference was defined at p < 0.05 (*, or #).

## Results

### PHB is differentially expressed in granulosa cells from different follicular stages

**Figure 1:**
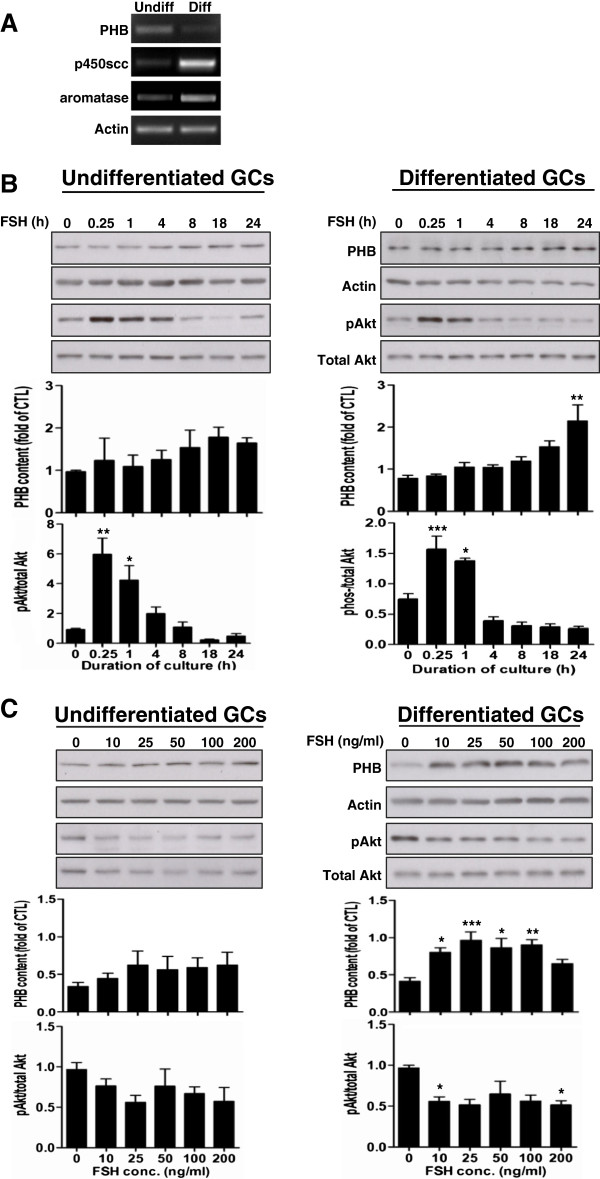
**The regulation of PHB by gonadotropin.** (**A**) Total RNAs of undifferentiated and differentiated granulosa cells were extracted and mRNA abundance of PHB was analyzed by PCR. p450scc and aromatase mRNA levels were assessed as differentiated marker and Actin was used as loading control. (**B**) Undifferentiated and differentiated granulosa cells were cultured with FSH (100 ng/ml) for designated time period and the contents of PHB, pAkt and total Akt were examined by Western blot. (**C**) Undifferentiated and differentiated granulosa cells were cultured with FSH (0–200 ng/ml) for 24 h. PHB, pAkt and total Akt contents were examined by Western blot. Representative immunoblots are shown and data are presented as mean ± SEM of three independent experiments. B-C, one-way ANOVA, followed by Bonferroni test. *, p < 0.05; **, p < 0.01; ***, p < 0.001 (compared with FSH = 0 or FSH at 0 h).

To examine whether PHB is expressed *in vivo* in a follicular stage-dependent manner, differentiated and undifferentiated granulosa cells were collected and the gene expression of PHB was analyzed by traditional PCR. As shown in Figure 1A, differentiated granulosa cells exhibited increased expression of *p450scc* and *aromatase*, steroidogenic enzymes known to be associated with granulosa cell differentiation. The *PHB* expression in differentiated granulosa cells was lower than that in undifferentiated granulosa cells.

### FSH upregulates PHB content in differentiated but not undifferentiated granulosa cells

To explore whether FSH regulates PHB expression *in vitro*, undifferentiated and differentiated granulosa cells were cultured with FSH (100 ng/ml) for designated time (0–24 h) and the contents of PHB in two types of cells were examined. As shown in Figure 1B, FSH had no effect on PHB content in undifferentiated granulosa cells (p > 0.05). In the contrast, FSH up-regulated PHB expression in differentiated granulosa cells (p < 0.001). While there was an apparent gradual increase in PHB content with the duration of culture in the presence of FSH, significant upregulation was not evident until 24 h. Both undifferentiated (p < 0.0001) and differentiated (p < 0.001) granulosa cells exhibited a rapid increase in phosphorylated Akt content; however, Akt activation in the former was stronger (6-fold vs. 2-fold change at 0.25h) and sustained longer (over basal level at 4-8h) compared with that in the latter.

We then examined whether FSH regulates PHB contents in differentiated granulosa cells in a concentration-dependent manner. Undifferentiated and differentiated granulosa cells were cultured with different FSH concentration (0–200 ng/ml) for 24 h and the content of PHB at the two follicular stages was examined. As shown in Figure 1C, FSH had no effect on PHB content in undifferentiated granulosa cells (p > 0.05); however FSH significantly up-regulated the content of PHB (p < 0.001) at a concentration range of 10–100 ng/ml in differentiated granulosa cells. There was no significant difference between the FSH (200 ng/ml) and the control groups. Since we have reported that Akt and PHB could regulate each other [23], we also assessed the phosphorylated and total Akt levels in this experiment. As shown in Figure 1C, although there was a trend of decrease, FSH didn’t significantly reduce Akt phosphorylation in undifferentiated granulosa cells (p > 0.05). In the contrast, phosphorylated Akt levels in granulosa cells in differentiated granulosa cells decreased in the presence of FSH (Figure 1C, p < 0.05), which was reversely related to PHB contents.

### Exogenous PHB suppresses staurosporine-induced caspase-3 cleavage and apoptosis in undifferentiated, but not differentiated, granulosa cells

**Figure 2:**
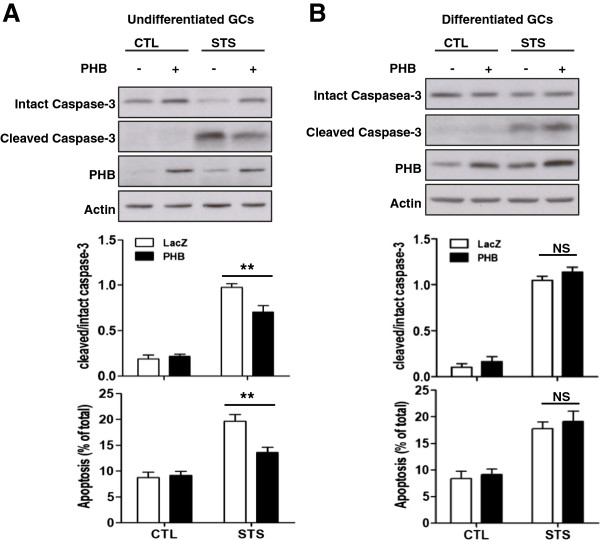
**Effect of exogenous PHB on STS-induced caspase-3 cleavage and apoptosis.** Undifferentiated (**A**) and differentiated (**B**) granulosa cells were infected with adenoviral-PHB (MOI = 40, adenoviral-lacZ as control) for 24 h and then cultured with STS (1 μM, 2 h). The contents of cleaved, intact caspase-3 and PHB were examined by Western blot and the apoptosis rate of granulosa cells were assessed by nuclear morphology (Hoechst staining). Data are presented as mean ± SEM of three independent experiments and analyzed by two-way ANOVA and subsequently by Bonferroni post hoc test. **, p < 0.01 (compared with LacZ). NS: not significant.

Although PHB has been reported to be anti-apoptotic in undifferentiated granulosa cells [19,20], whether it plays a similar role in granulosa cells after differentiation is not known. To address this question, comparison experiments were performed using undifferentiated and differentiated granulosa cells. Granulosa cells were infected with adenoviral-PHB (MOI = 40, adenoviral-LacZ as control, 24 h) to over-express or knockdown PHB, and then cultured with the apoptosis inducer staurosporine (STS, 1 μM, 2 h). As shown in Figure 2A, whereas PHB over-expression had no effect on basal level of apoptosis in undifferentiated granulosa cells, it suppressed STS-induced caspase-3 cleavage (Figure 2A, PHB, p < 0.05; STS, p < 0.0001; PHB × STS, p < 0.05) and apoptosis (PHB, p < 0.05; STS, p < 0.0001; PHB × STS, p < 0.05). In contrast, overexpression of PHB in differentiated granulosa cells had no obvious effect on STS-induced caspase-3 cleavage and apoptosis (Figure 2B), suggesting that PHB is anti-apoptotic during preantral follicular growth but this property is lost as the cells differentiate during follicle transition into the antral stage.

### Knockdown of PHB increases staurosporine-induced caspase-3 cleavage and apoptosis in undifferentiated, but not differentiated, granulosa cells

**Figure 3:**
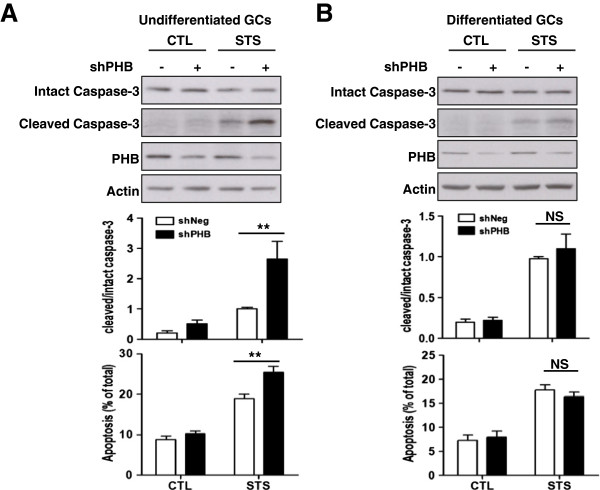
**Effect of PHB knockdown on STS-induced caspase-3 cleavage and apoptosis.** Undifferentiated (**A**) and differentiated (**B**) granulosa cells were infected with adenoviral-shPHB (MOI = 10, adenoviral-shNeg as control) for 48 h and then cultured with STS (1μM, 2 h). The contents of cleaved, intact caspase-3 and PHB were examined by Western blot and the apoptosis rate of granulosa cells were assessed by nuclear morphology (Hoechst staining). Data are presented as mean ± SEM of three independent experiments and analyzed by two-way ANOVA and subsequently by Bonferroni post hoc test. **, p < 0.01 (compared with LacZ). NS: not significant.

Similar experiments were performed using adenoviral-shRNA to knockdown PHB in granulosa cells derived from two preparations. As shown in Figure 3, STS-induced caspase-3 cleavage and apoptosis were enhanced after knockdown of PHB in undifferentiated granulosa cells (Figure 3A, for cleaved caspase-3, shPHB, p < 0.01; STS, p < 0.0001; shPHB × STS, p < 0.05. For apoptosis, shPHB, p < 0.01; STS, p < 0.0001; shPHB × STS, p < 0.05). However, there was no effect of PHB knockdown on STS-induced caspase-3 cleavage and apoptosis in differentiated granulosa cells (Figure 3B).

### Differentiated granulosa cells are more responsive to FSH in steroids production compared with undifferentiated granulosa cells

**Figure 4:**
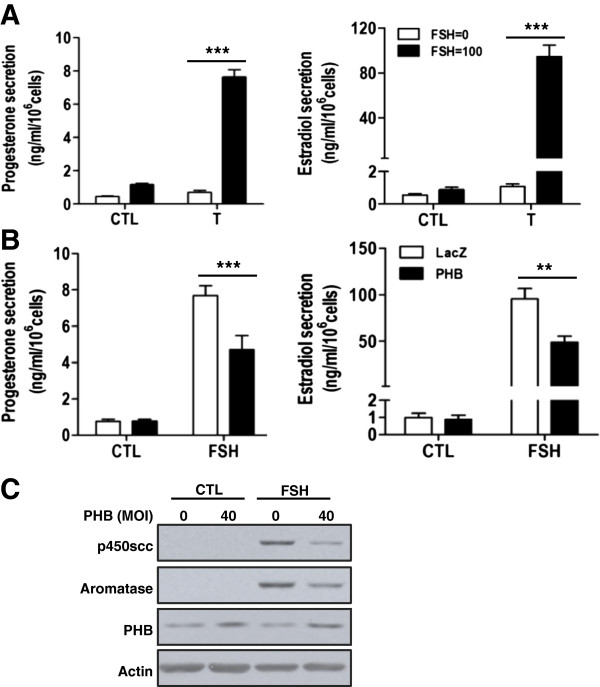
**Influence of PHB on FSH-induced steroid production.** (**A**) Differentiated granulosa cells were cultured with FSH (100 ng/ml) ± T (0.5 μM) for 24 h and the levels of progesterone and estradiol in the spent medium were measured by EIA. Data are presented as mean ± SEM of four independent experiments and analyzed by two-way ANOVA and subsequently by Bonferroni post hoc test. ***, p < 0.001 (compared with LacZ). (B-C) Differentiated granulosa cells were infected with adenoviral-PHB (MOI = 40, adenoviral-LacZ as control) for 24 h and then cultured with FSH (100 ng/ml, 24 h) in the presence of T (0.5 μM). Progesterone and estradiol in the spent medium (**B**) were measured by EIA. Data are presented as mean ± SEM of four independent experiments and analyzed by two-way ANOVA and subsequently by Bonferroni post hoc test. **, p < 0.01; ***, p < 0.001 (compared with CTL). (**C**) The expression of p450scc and aromatase was examined by Western blot. Representative immunoblots from three replicate experiments are shown.

We have previously reported that PHB suppressed FSH-induced estradiol and progesterone secretion and p450scc/aromatase expression in undifferentiated granulosa cells [23]. As the regulatory role of PHB in granulosa cell apoptosis is dependent on the state of cellular differentiation, we then examined whether it regulate steroidogenesis differently in differentiated granulosa cells by comparing the steroidogenic responsiveness of granulosa cells at the two differentiative states. Undifferentiated and differentiated granulosa cells from two preparations were cultured with FSH (0–100 ng/ml) ± testosterone (T, 0.5 μM, 24 h), which served as a substrate of aromatase, and then the levels of estradiol and progesterone in spent medium were measured. We have previously demonstrated that T enhanced FSH-induced progesterone and estradiol secretion in undifferentiated granulosa cells [23]. We observed that FSH-induced progesterone production in these cells was dramatically increased in the presence of T (5.49 ± 0.4 vs. 0.63 ± 0.13 ng/ml) and the estradiol secretion exhibited similar effect (48.7 ± 5.7 vs. 0.91 ± 0.16 ng/ml). We concomitantly tested the effect of T and FSH on differentiated cells (Figure 4A) and observed that T also enhanced FSH-stimulated progesterone (7.79 ± 0.63 vs. 1.22 ± 0.98 ng/ml) and estradiol secretion (94.4 ± 10.23 vs. 0.88 ± 0.14 ng/ml) in differentiated granulosa cells (Figure 4A, for progesterone: FSH, p < 0.001; T, p < 0.001; FSH × T, p < 0.001. for estradiol: FSH, p < 0.001; T, p < 0.001; FSH × T, p < 0.001). The basal levels of estradiol and progesterone at the two cellular differentiative states were similar; however production of these steroids in the presence of FSH and T was about 2-fold higher in differentiated granulosa cells than in undifferentiated granulosa cells.

### PHB suppresses FSH-induced steroid production in both undifferentiated and differentiated granulosa cells

Next we examined whether PHB suppresses FSH-induced steroidogenesis in granulosa cells and if its response is dependent on the differentiated state of the cells. Granulosa cells from two preparations were infected with adenoviral-PHB (adenoviral-LacZ as control) and then cultured with FSH (0–100 ng/ml) in the presence of T (0.5 μM) for 24h. The levels of estradiol and progesterone in spent medium were measured by EIA. Exogenous PHB suppressed FSH-induced steroid production and the expression of steroidogenic enzymes p450scc and aromatase in undifferentiated granulosa cells [23]. FSH-induced progesterone production in these cells was dramatically suppressed by exogenous PHB (5.45 ± 0.47 vs. 3.14 ± 0.35 ng/ml) and the estradiol secretion exhibited similar effect (55.6 ± 5.0 vs. 21.76 ± 7.72 ng/ml). Concomitant studies with differentiated granulosa cells indicate that PHB similarly inhibited FSH-induced progesterone (7.70 ± 0.52 vs. 4.70 ± 0.78 ng/ml) and estradiol (95.56 ± 11.17 vs. 48.96 ± 6.7 ng/ml) secretion (Figure 4B, for progesterone: PHB, p < 0.01; FSH, p < 0.001; PHB × FSH, p < 0.01. for estradiol: PHB, p < 0.05; FSH, p < 0.001; PHB × FSH, p < 0.05) *in vitro.* The contents of p450scc and aromatase induced by FSH were also suppressed by PHB (Figure 4C).

## Discussion

In the present study, we have demonstrated the distinct roles of PHB in STS-induced apoptosis and FSH-induced steroidogenesis in granulosa cells at different stages of follicular development and thus at different cellular differentiative states. FSH regulates PHB expression in differentiated but not undifferentiated granulosa cells *in vitro*. PHB is anti-apoptotic and a suppressor of steroidogenesis in undifferentiated granulosa cells, while it loses its role in regulating apoptosis but maintains its latter action as the cells differentiate. These findings suggest that suppressive roles of PHB in apoptosis and steroidogenesis are precisely regulated in a follicular stage-dependent manner. The role of PHB in the regulation of apoptosis, steroidogenesis as well as proliferation [11,12], the dysregulation of ovarian follicular growth and steroidogenesis in polycystic ovarian syndrome (PCOS) [26-28], together with the observations that the expression of PHB increased in a chronically androgenized rat PCOS model [23] suggest a possibility that dysregulation of PHB may be relevant to the etiology of this syndrome.

The main observation in present study is that the anti-apoptotic action of PHB is evidenced at the preantral follicle stage of development, one often referred to as the “penultimate stage” when the destiny of the follicle (continued growth versus atresia) is determined. This observation also raise the interesting possibility that PHB may play an important role in deciding the fate of the granulosa cells as the follicles transition from the preantral to early antral stage. With regard to the regulation of apoptosis, PHB may execute its anti-apoptotic role by down-regulating caspase-3 cleavage and inhibiting cytochrome c release from mitochondria [20]. The loss of inhibition on apoptosis in differentiated granulosa cells may due to increased X-linked inhibitor of apoptosis protein (XIAP) expression and elevated Akt phosphorylation in these cells [29] known to inhibit apoptosis, which may modulate the action of PHB. However, we cannot rule out the possibility that PHB inhibits apoptosis via regulating the activity of transcriptional factors involved in apoptosis, as demonstrated by Fusaro *et al.* that PHB protects cancer cells from camptothecin-induced apoptosis via suppressing E2F1-mediated transcriptional activity [30]. While the different transcriptional factors targeted by PHB may switch off its anti-apoptotic function, this hypothesis needs further investigation.

In contrast, the participation of PHB in the control of steroidogenesis in both undifferentiated and differentiated granulosa cells is consistent with important intraovarian regulatory role of steroids during follicular development and the involvement of PHB in the control of the steroidogenic processes. The mechanism by which PHB acts as a steroidogenesis-suppressor or anti-apoptotic factor during follicular development and the physiological signal that drives these responses are unknown. PHB is shown to be regulated by the novel adipokine chemerin *in vitro* and it mediates the suppressive role of chemerin on FSH-induced steroidogenic enzyme expression in undifferentiated granulosa cells [23]. Because PHB is a co-activator or co-repressor of distinct transcriptional factors (p53, E2F, ERα) [7,17,18] and we have observed an interaction of PHB and NR5a1/2 in freshly isolated rat granulosa cells (Additional file 1: Figure S1), it is possible that PHB suppresses steroidogenesis via acting as a co-repressor of transcriptional factors targeting steroidogenic enzymes, such as nuclear receptors NR5a1/NR5a2, C/EBP [31-34]. Further studies on promoter activity, DNA mutagenesis and protein-protein interaction assay are needed to test this possibility. However, we cannot rule out the possibility that PHB may regulate the mRNA expression of FSH receptor.

Besides PHB, other proteins have also been reported to be differentially regulated and play various functions in ovarian cells from different follicular stages. Plasminogen activator plays a crucial role in the dynamic tissue remodeling during follicular development and ovulation [35,36]. Its activity is increased by FSH, and inhibited by transforming growth factor α, in undifferentiated granulosa cells but decreased in differentiated ones [37,38]. Another example is C-type natriuretic peptide (CNP), which binds to its receptor and promotes preantral follicle growth via stimulating the cGMP release in undifferentiated granulosa cells; however CNP is unable to increase cGMP level in differentiated granulosa cells [39]. The cell differentiation-dependent regulation of intra-ovarian and intracellular factors may facilitate the precise control of granulosa cell fate and function during follicular development.

Although the functions of PHB are reported to regulate many cellular processes in various cell types, how its expression is regulated is largely unknown. The regulation of PHB by gonadotropin in the literature is controversial. We demonstrated that gonadotropin *in vivo* reduced PHB mRNA abundance; however others reported that PHB mRNA levels and protein contents are not altered [40] or higher after gonadotropin treatment [21]. While the reasons for these apparent differences are not immediately clear, whether differences in the dosages of gonadotropin used and/or in the methods of granulosa cell isolation could account for the different outcome, remains to be determined. In this context, our results also indicated that high concentration of FSH (200 ng/ml) failed to increase PHB expression *in vitro* whereas lower concentration did (Figure 1C). The effect of high doses of FSH or other molecules on gene expression has been well documented and an effective negative feedback mechanism to precisely control gene expression may be operational. FSH receptor is desensitized and down-regulated by long exposure of high dose of FSH [41-43], accompanied by reduced *cyp19* expression and estradiol production in granulosa cells [43]. In addition, anti-Müllerian hormone, known to inhibit FSH-induced aromatase expression, is also up-regulated by low but down-regulated by high dose of FSH [43]. Low dose of AMH stimulates inhibin B level in human granulosa cells while the opposite was true with higher dosages [44].

The mechanism by which FSH regulates PHB expression is unclear. It is known that FSH acts through multiple signaling pathways, including cAMP/PKA, PI3K/Akt and MAPK, and via various transcriptional factors, such as forkhead box O1, cAMP regulatory element binding protein and specific protein 1 (Sp1) [45-48]. In addition to putative binding sites for CCAAT/enhancer-binding protein (C/EBP) and insulin response element in the promoter of PHB [49], there are other DNA binding elements such as E2F, GATA, ER and Sp1 as predicted by the transcription element search system. It is possible that the transcription factors maintaining PHB expression in undifferentiated granulosa cells is switched to others in differentiated granulosa cells due to the distinct cellular contents. However, the particular transcription factor involved in the regulation of PHB in granulosa cells at the two stages of cellular differentiation needs to be further investigated.

It is well known that FSH stimulates Akt phosphorylation in ovarian cells [45]. Our present studies extent these findings and show that the rapid increase of phosphorylated Akt content in response to FSH is dependent on the state of cellular differentiation. Since the phosphorylated Akt content is a consequence of both kinase and phosphatase activities [50], it was of interest to determine whether this signal is removed with a different efficiency between different state of differentiation and whether the decreased pAkt levels are correlated with increased PHB contents. Our results indicated that the efficiency at which the PI3K-Akt signaling pathway is turned on and off by FSH is also dependent on the state of cellular differentiation and may be related to the action of PHB. The reverse correlation of PHB and pAkt content was supported by a recent finding that PHB and Akt could regulate the expression of each other [23].

In our culture system, both undifferentiated and differentiated granulosa cells exhibited a robust steroidogenic response to FSH. Basal and FSH-induced estradiol secretion in undifferentiated granulosa cells were lower than those in differentiated granulosa cells in the presence of testosterone, which could be due to higher basal levels of p450scc and aromatase induced by gonadotropin with increased granulosa cell differentiation (this study, [51]). Testosterone was added in the culture as the substrate of aromatase, which is commonly used in the studies on FSH-induced estrogen production [45,52,53]. It is also possible that testosterone not only acted as a substrate in granulosa cells, but also augmented the action of FSH on the production of progesterone and estradiol [52,54,55].

## Conclusion

Our findings demonstrate that PHB expression is regulated by FSH in a follicular stage-dependent manner *in vitro* and the roles of PHB as an anti-apoptotic factor and in the regulation of steroidogenesis are dependent on the differentiation status of granulosa cells. It is an inhibitor of steroidogenesis in both undifferentiated and differentiated granulosa cells, but is anti-apoptotic in undifferentiated granulosa cells. This study significantly improves our understanding of the role of PHB in the ovary although the mechanism by which PHB suppresses apoptosis and steroidogenesis and the factors involved in the regulation of PHB remains to be investigated.

## Abbreviations

PHB: Prohibitin; STS: Staurosporine; eCG: Equine chronic gonadotropin; DES: Diethylstilbestrol; ECL: Enhanced chemiluminescent; MOI: Multiplicity of infection; EIA: Enzyme immunoassay; T: Testosterone; p450scc: P450 side-chain cleavage enzyme.

## Competing interests

The authors declare that they have no competing interests.

## Authors’ contributions

QW performed the experiments, prepared the data and drafted the manuscript. AL and BKT are co-mentors, provided input of studies and edited the manuscript. All authors read and approved the final manuscript.

## Supplementary Material

Additional file 1: Figure S1 Interaction of PHB and NR5a1/2. Granulosa cells were collected from eCG-primed rats and then lyzed in IP lysis buffer. Endogenous PHB in 500 μg cell lysate was immunoprecipitated with 2 μg mouse anti-PHB antibody (normal mouse IgG as control), subjected to 15% SDS-PAGE and probed with the antibodies targeting PHB, NR5a1 and NR5a2, respectively. Darker bands in Western blot indicate the heavy or light chain of IgG. Click here for file
