# Supplementary figures and images for: Follicular stage-dependent regulation of apoptosis and steroidogenesis by prohibitin in rat granulosa cells
Source: J Ovarian Res. 2013 Apr 8;6:23. doi: 10.1186/1757-2215-6-23 (PMC3635931; doi:10.1186/1757-2215-6-23)

## Slide 1
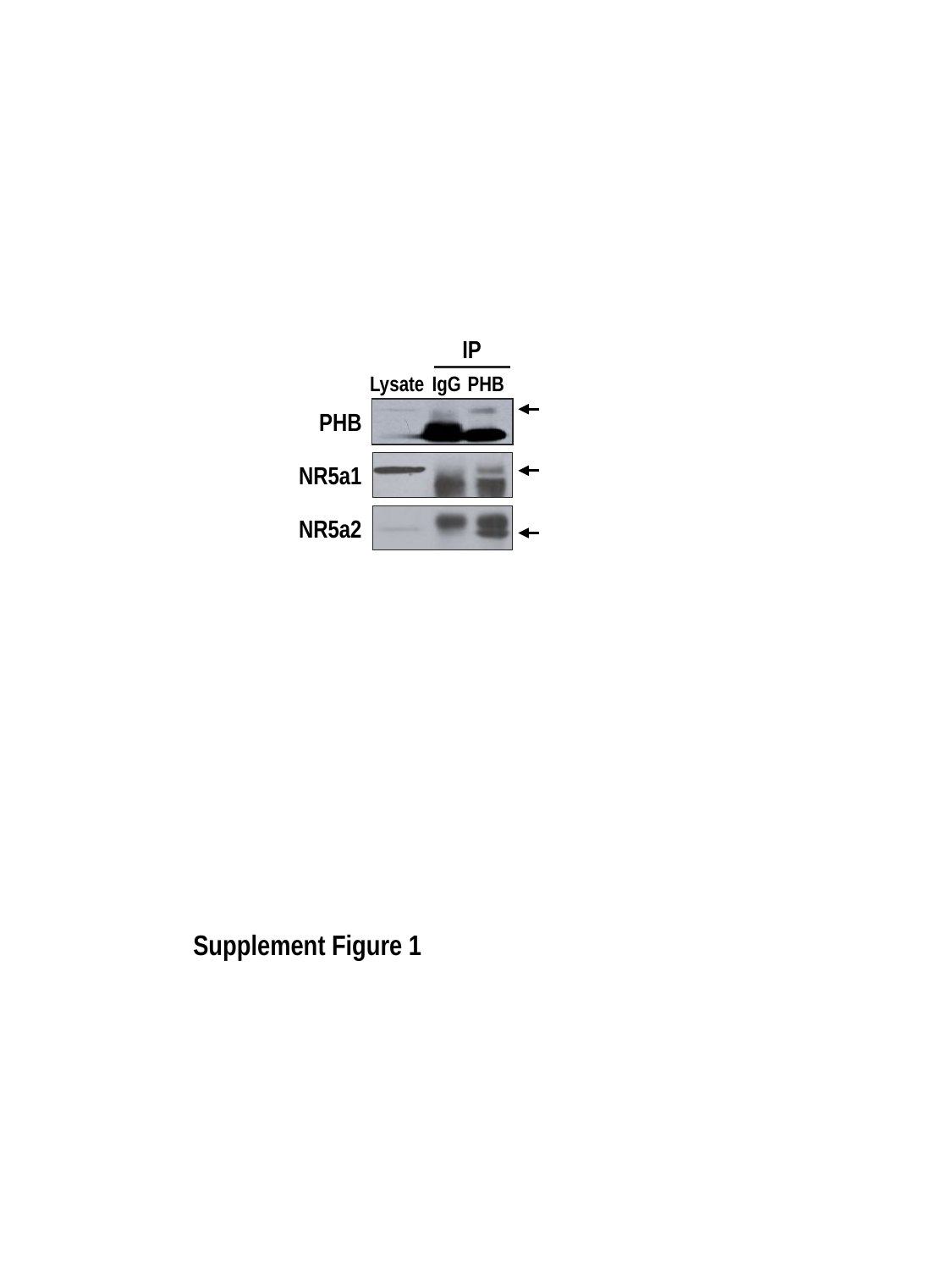

IP
Lysate
IgG
PHB
PHB
NR5a1
NR5a2
Supplement Figure 1

Supplement: Additional file 1: Figure S1 — Interaction of PHB and NR5a1/2. Granulosa cells were collected from eCG-primed rats and then lyzed in IP lysis buffer. Endogenous PHB in 500 μg cell lysate was immunoprecipitated with 2 μg mouse anti-PHB antibody (normal mouse IgG as control), subjected to 15% SDS-PAGE and probed with the antibodies targeting PHB, NR5a1 and NR5a2, respectively. Darker bands in Western blot indicate the heavy or light chain of IgG. [file 1757-2215-6-23-S1.pptx]
